# Relationship between *ITPA* polymorphisms and hemolytic anemia in HCV-infected patients after ribavirin-based therapy: a meta-analysis

**DOI:** 10.1186/s12967-015-0682-y

**Published:** 2015-10-06

**Authors:** Daniel Pineda-Tenor, Mónica García-Álvarez, María A. Jiménez-Sousa, Sonia Vázquez-Morón, Salvador Resino

**Affiliations:** Servicio de Laboratorio Clínico, Hospital Universitario de Fuenlabrada, Fuenlabrada, Madrid, Spain; Unidad de Infección Viral e Inmunidad, Centro Nacional de Microbiología, Instituto de Salud Carlos III, Majadahonda-Pozuelo, km 2.2, Majadahonda, 28220 Madrid, Spain

**Keywords:** ITPA, Hemolytic anemia, SNPs, Ribavirin, Chronic hepatitis C, HCV therapy

## Abstract

**Background:**

There is growing evidence that variations in the gene encoding inosine triphosphate pyrophosphohydrolase (ITPase), known as *inosine triphosphatase (ITPA)*, are related to hemolytic anemia, which is frequently observed among hepatitis C virus (HCV)-infected patients receiving ribavirin (RBV)-based therapy. We performed a meta-analysis of all eligible studies assessing *ITPA* gene polymorphisms related to RBV-induced hemolytic anemia in HCV-infected patients published in PubMed, Embase and the Cochrane library prior to the end of 2014.

**Methods:**

Three outcomes were evaluated: (1) hemoglobin decline, (2) severe anemia, and (3) RBV dose reduction or treatment discontinuation. Pooled odds ratio (OR) and 95 % confidence interval (95 % CI) were estimated by either fixed or random effects models.

**Results:**

Twenty-nine studies were selected from the literature search: 20 references involving 6533 individuals for hemoglobin decline, 13 references on 3764 patients for severe anemia, and 16 references on 3918 patients for RBV dose reduction or discontinuation. Significant associations with hemoglobin decline were found for rs1127354 CC [OR = 12.84 (95 % CI 7.44; 22.17)], rs7270101 AA [OR = 3.41 (95 % CI 2.08; 5.59)] and rs6051702 AA [OR = 4.43 (95 % CI 2.80; 7.00)] genotypes. Moreover, significant associations with hemoglobin decline were also found for absent [OR = 6.01 (95 % CI 4.84; 7.46)] and mild [OR = 4.68 (95 % CI 2.83; 7.74)] ITPase deficiency haplotypes. The *ITPA* rs1127354 CC genotype and absent ITPase deficiency haplotype were also associated with severe anemia {[OR = 7.77 (95 % CI 5.03; 12.00)] and [OR = 4.79 (95 % CI 1.69; 13.56)], respectively}. Additionally, the rs1127354 CC genotype showed significant association with RBV dose reduction or stopping treatment (OR = 2.24; 95 % CI 1.79; 2.81).

**Conclusions:**

*ITPA* polymorphisms increase the likelihood of developing hemolytic anemia for HCV-infected patients on RBV-based therapy, particularly rs1127354 CC and rs7270101 AA genotypes, suggesting the utility of screening for *ITPA* polymorphisms to avoid hematological toxicity and increase adherence to RBV-based therapy.

**Electronic supplementary material:**

The online version of this article (doi:10.1186/s12967-015-0682-y) contains supplementary material, which is available to authorized users.

## Background

For many years, pegylated interferon-alpha (pegIFNα) plus ribavirin (RBV) combination therapy has been the standard treatment for hepatitis C virus (HCV) infection, but the side-effects have made the therapy arduous for many patients [1]. Recently, new direct-acting antivirals (DAAs) have improved the response rate, particularly in difficult-to-treat patients infected with HCV genotypes (GT) 1 or 4, and have made adverse effects less common [2, 3]. Nevertheless, due to the high cost of DAAs, only patients with advanced liver disease will be initially treated with DAAs according to the new guideline [4]. Moreover, pegIFNα/RBV therapy remains an effective antiviral treatment option for patients infected with GT2 and GT3, and its relatively low cost is an advantage. Furthermore, the use of triple therapy with pegIFNα/RBV and DAAs (e.g. simeprevir, sofosbuvir) is still recommended as well as the use of DAAs with RBV only in certain subgroups of patients [2–4]. Thus, RBV continues to maintain an important role in HCV therapy even with the introduction of DAAs [2–4].

Hemolytic anemia is a common side-effect in HCV-infected patients on pegIFNα/RBV therapy, affecting up to 30 % of patients, which requires close monitoring of hemoglobin and dose modification in up to 15 % of patients [5]. RBV-induced anemia primarily results in the reduction of adenosin triphosphate (ATP) levels in erythrocytes, affecting ATP-dependent oxidative metabolism [6, 7]. Genetic variations in the *inosine triphosphatase (ITPA)* gene, which encodes an inosine triphosphate pyrophosphohydrolase (ITPase), are associated with protection from hemolytic anemia during pegIFNα/RBV therapy [8]. These *ITPA* variants affect ITPase functionality, causing a drop in its activity, resulting in an accumulation of inosine triphosphate (ITP) in erythrocytes and the prevention of oxidative stress [6, 9].

Initially, two *ITPA* variants (rs1127354 and rs7270101) were found to be associated with protection against hemolytic anemia during pegIFNα/RBV therapy [8, 10]. Single nucleotide polymorphisms (SNPs) at both of these locations result in functional variants that code for a missense mutation in exon 2 (rs1127354, P32T) or alter a splice site (rs7270101) [11, 12]. Homozygosity for these *ITPA* minor alleles leads to ITPase deficiency and a strong accumulation of ITP in erythrocytes, which is associated with lower RBV-toxicity. The *ITPA* rs6051702 C minor allele, a more common variant, has also been associated with protection from anemia [8].

In recent years, a large number of articles about *ITPA* polymorphisms and RBV-induced anemia have been published, although conflicting results have been reported. For that reason, our aim was to carefully analyze the relationship between *ITPA* polymorphisms and hemolytic anemia in HCV-infected patients on RBV-based HCV therapy by conducting a meta-analysis of all eligible studies published to date (December 31, 2014).

## Methods

### Search strategy and study selection

Relevant studies were identified by searching Pubmed, Embase and the Cochrane Library from inception through December 31, 2014; using the following terms: (“hepatitis C” or “HCV” or “chronic hepatitis C”), (“ITPA” or “inosine triphosphatase”) (“SNP” or “polymorphism”). No language restrictions were applied. The meta-analysis was conducted following guidelines from Sutton et al. [13], and the data were reported in accordance with the Preferred Reporting Items for Systematic Reviews and Meta-Analyses (PRISMA) guidelines [14].

We applied strict inclusion and exclusion criteria before reviewing the studies and extracting the data:

- *Inclusion criteria* (1) patients infected with HCV or HCV/human immunodeficiency virus (HIV) coinfection; (2) any SNP located within or near the *ITPA* gene (described in two or more articles); (3) HCV treatment-based RBV alone or in combination with pegIFNα (2a or 2b) (combined or not with DAAs); (4) available data on at least one outcome.
- *Exclusion criteria* (1) coinfection with hepatitis B virus; (2) treatment duration of less than 12 weeks or no treatment; (3) absent or inadequate information about treatment, study population, HCV status, or not enough information to calculate the odds ratio (OR) and 95 % confidence intervals (95 % CI); (4) studies with sample size less than 40 subjects; (5) reviews, editorials, letters, chapters, conference abstracts or clinical case reports.

In order to select the candidate studies, we screened the title and abstract of each publication. When articles fulfilled the inclusion criteria, we examined the full text and extracted data from the study. When studies included several subgroups and some of them did not fulfil the inclusion criteria, we only incorporated into the meta-analysis those subgroups that did meet the inclusion criteria. When more than one article studying the same cohort was found, only the study with the most extensive cohort was reviewed, excluding the remaining overlapping studies or data.

Two authors (DPT and MGA) performed the literature search and the study selection separately.

### Data extraction

Data were extracted independently by two investigators (DPT and MGA) and then cross-checked. When data were unclear or required assumptions to be made, other investigators (MAJS and SR) were consulted so that a consensus could be reached before recording an entry in the database. Authors of included studies were contacted when the data were not explicitly reported or any clarification was needed.

### Outcome variables

Three outcome variables were evaluated: (1) *Hemoglobin decline* a decrease in hemoglobin of more than 2 or 3 g/dL [8, 10, 15–32]; (2) *Severe anemia* hemoglobin levels less than 8.5, 8.9, 10 or 10.5 g/dL [8, 18, 20, 23, 24, 26, 27, 30–35]; (3) *Ribavirin dose reduction or discontinuation of treatment* that resulted from significant anemia defined by several cut-offs (8.5, 10 or 12 g/dL) or physician’s criteria [10, 15–18, 22, 27, 30, 34–41].

### *ITPA* polymorphisms

The following *ITPA* polymorphisms and haplotypes were included in the analyses:

1. rs1127354 (C>A): missense variant in exon 2 resulting in a proline-to-threonine substitution (P32T) [8]. NCBI SNP database: http://www.ncbi.nlm.nih.gov/projects/SNP/snp_ref.cgi?rs=1127354.
2. rs7270101 (A>C): splice-altering SNP located in the second intron [8]. NCBI SNP database: http://www.ncbi.nlm.nih.gov/projects/SNP/snp_ref.cgi?rs=7270101.
3. rs6051702 (A>C): non-functional variant localized in a non-coding region adjacent to the *ITPA* gene (20p13) [8]. NCBI SNP database: http://www.ncbi.nlm.nih.gov/projects/SNP/snp_ref.cgi?rs=6051702.
4. rs1127354/rs7270101 haplotype: ITPase deficiency ranging from absent (−) (representing wild-type activity) to mild (+), moderate (++) or severe (+++) (Additional file 1: Box 1) [8, 10].

The SNPs studied were in agreement with Hardy–Weinberg equilibrium (p > 0.05), estimated by the Chi squared test.

### Quality assessment

Two investigators (MGA and DPT) independently evaluated the study quality using an evaluation system modified from the Newcastle–Ottawa Scale. A description of the adapted methodological quality criteria is available in Additional file 1: Box 2. The full score was 20 stars, and a high-quality study was defined as a study with 15 or more stars.

### Statistical analysis

All analyses were performed using Stata software (version 11.0; Stata Corporation, College Station, TX, USA). All p values <0.05 were considered significant.

Overall, meta-analysis was performed only when two or more articles studying the same outcome were available. In all analyses, pooled OR and 95 % CI were calculated. The significance of the pooled OR was calculated by the Z test. The study heterogeneity was assessed using the Cochran’s Q statistic and I^2^ statistic, considering a Q statistic p < 0.1 or I^2^ > 50 % as significant heterogeneity. A fixed effect model (a traditional Mantel–Haenszel method) was used for homogeneous studies. When significant heterogeneity existed, a random effect model was applied (DerSimonian and Laird method) and a Galbraith plot was used to detect possible outliers of the heterogeneity. In addition, when heterogeneity was detected, meta-regression analysis was also performed with the aim of defining the potential effect of the covariates on the outcome variables. The regression coefficients obtained describe how the hemoglobin decline, severe anemia or ribavirin dose reduction changed with each unit increase in the covariate. Significance of the linear relationship was identified by the p value. The covariates analyzed were: cut-off, time of analysis, sex, age, racial descent, HCV-genotype, HCV therapy and HIV coinfection.

Publication bias was assessed by Begg’s funnel plot and the Egger linear regression test which detects funnel plot asymmetry. Publication bias was assumed to exist when the Egger test reported a p < 0.05. The sensitivity analyses were also conducted to assess the consistency of results and to investigate the influence of one study on the overall meta-analysis. It was carried out by sequential omission of individual studies.

## Results

### Search results

**Fig. 1:**
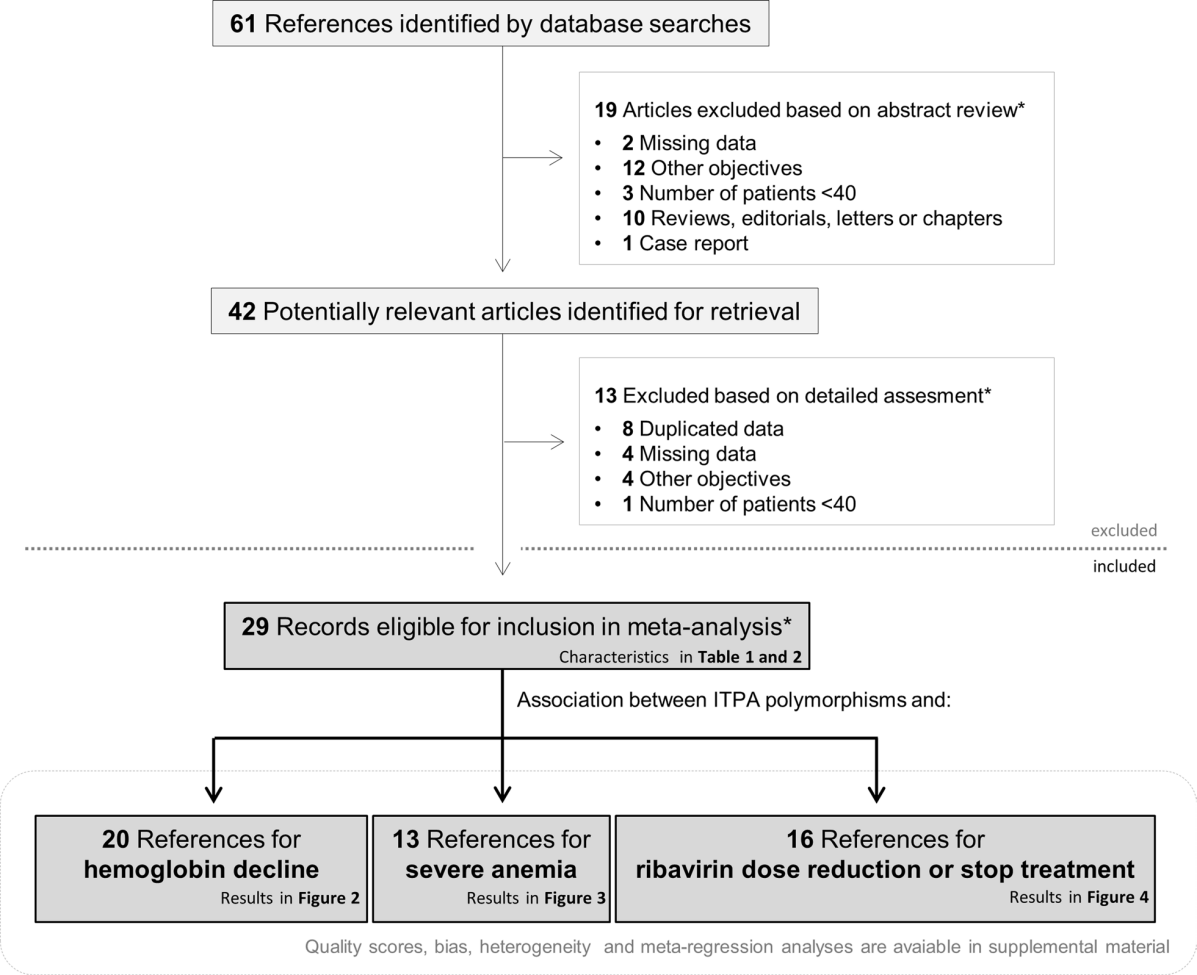
Flowchart showing the selection of articles included in the meta-analysis. *Asterisk* several studies were eligible for different categories

The search strategy yielded 61 entries, 42 of which were considered to have potential value and the full texts were retrieved for detailed evaluation (Fig. 1). After exclusion based on detailed assessment, 29 studies were eligible for inclusion (20 for hemoglobin decline, 13 for severe anemia and 16 for RBV dose reduction or discontinuation of treatment meta-analysis). Seventeen of the 29 studies were included in more than one analysis.

### Article characteristics

The main characteristics of the included studies from 2010 to 2014 are summarized in Table 1: 533 individuals for hemoglobin decline, 3764 patients for severe anemia, and 3918 patients for RBV dose reduction or discontinuation of treatment. Data were collected from several countries with different ethnicities and HCV genotypes. Five references included HCV/HIV-coinfected patients, and three studies included HCV therapy based in PegIFN/RBV plus telaprevir. The specific characteristics for each outcome are showed in Table 2. Several cut-offs for the outcome definition were employed and various times of analysis were used.

**Table 1:** General characteristics of studies included in our meta-analysis for hemoglobin decline, severe anemia and ribavirin dose reduction or discontinuation

Year	First author	Design	N	Age (years)	Gender (% male)	Country	Ethnicity (%)	HCV-GT	GT1/4 (%)	HCV therapy	HIV (% on cART)
2010	Fellay [8]	GWAS	1286	47.3	61.74	USA	C (76.8), H (7.8), AA (15.4)	1	100	Peg- + RBV	No
2010	Thompson [10]	GWAS	318	48.5	65	USA	C (55), AA (45)	1	100	Peg-α2a + RBV	No
2010	Sakamoto [15]	Cross-sectional	474	57.2	55.7	Japan	A (100)	1b, 2a/b, 3a	NR	Peg-α2a/2b + RBV	No
2011	Thompson [16]	Retrospective	238	52	59	Italy, USA	C (100)	2, 3	0	Peg-α2b + RBV	No
2011	Azakami [17]	Retrospective	1002	58	53.9	Japan	A (100)	1, Others	NR	Peg-α2b + RBV	No
2011	Chayama [36]	NR	94	57	55.3	Japan	A (100)	1	100	Peg-α2b + RBV + TPV	No
2011	Kurosaki [18]	Retrospective	132	57.5	37.9	Japan	A (100)	1b	100	Peg-α2a/2b + RBV	No
2011	Rallón [19]	Retrospective	74	43	74	Spain	NR	1, 2, 3, 4	87	Peg-α2a/2b + RBV	Yes (NR)
2012	Nishimura [20]	NR	176	62	61.5	Japan	A (100)	1, 2	NR	Peg-α2a/2b + RBV	No
2012	Naggie [21]	Retrospective	161	42	75	Spain	C (100)	1, 2, 3, 4	70	Peg-α2a/2b + RBV	Yes (84)
2012	Domingo [22]	Prospective	73	46.8	58.9	Spain	NR	1, 3, 4	75.3	Peg-α2a + RBV	Yes (91.8)
2012	Osinusi [23]	NR	123	45.5	70.7	Germany, USA	C (58.5), AA (30.9), other (10.6)	1, 2, 3	NR	Peg-α2a/2b + RBV	Yes (83.1)
2012	Miyamura [37]	Retrospective	97	55.8	45.36	Japan	A (100)	1, 2	NR	Peg-α2a + RBV	No
2012	Vidal [33]	Prospective	113	40	74.3	Spain	NR	1, 2, 3, 4	61.6	Peg-α2a/2b + RBV	Yes (84.1)
2012	Tsubota [24]	Prospective	561	59.1	53.8	Japan	A (100)	1b	100	Peg-α2a/2b + RBV	No
2013	Rau [25]	Retrospective	216	NR	64.8	Switzerland	C (100)	1, 2, 3	NR	Peg-α + RBV	No
2013	Di Marco [26]	Prospective	233	58.7	64	Italy	NR	1, 2, 3	NR	Peg-α2b + RBV	No
2013	Ahmed [27]	Retrospective	102	32.5	88.2	Egypt	C (100)	1, 4	100	Peg-α2a/2b + RBV	No
2013	Ogawa [34]	Prospective	292	62	46.2	Japan	A (100)	1	100	Peg-α2b + RBV + TPV	No
2013	Scherzer [28]	Retrospective	308	43.9	60.1	Austria	C (100)	1	100	Peg-α2a + RBV	No
2013	Fujino [38]	Retrospective	120	NR	45.8	Japan	A (100)	1b	100	Peg-α2a/2b + RBV	No
2013	D’Avolio [29]	Retrospective	379	46	60.9	Italy	C (94.9), AA (5.1)	1, 2, 3, 4	56.2	Peg-α2a/2b + RBV	No
2013	Clark [30]	Prospective	193	NR	56.5	Italy	NR	1, 2, 3, 4	63.73	Peg-α2a + RBV	No
2013	Seto [39]	NR	60	49	68.3	Hong Kong	A (100)	6	0	Peg-α2a/2b + RBV	No
2013	Nakagawa [40]	Cross-sectional	300	57	51	Japan	A (100)	1, 2	NR	Peg- + RBV	No
2014	Matsuura [31]	NR	309	57	52	Japan	A (100)	1	100	Peg-α2a/2b + RBV	No
2014	Rembeck [41]	Retrospective	354	42	42	Scandinavian	C (100)	2, 3	0	Peg-α2a + RBV	No
2014	Aghemo [35]	Retrospective	69	57	67	Italy	NR	1	100	Peg-α2a/2b + RBV + TPV	No
2014	Hwang [32]	Cross-sectional	175	55.5	66.3	Taiwan	NR	1, others	63.4	Peg-α2a/2b + RBV	No

*A* asians, *AA* africans ascendence, *C* caucasians, *H* hispanics, *Hb* hemoglobin, *NR* not reported, *Peg-IFN* pegilated inferferon, *RBV* ribavirin, *TPV* telaprevir

**Table 2:** Specific characteristics of included studies for hemoglobin decline (A), severe anemia (B) and ribavirin dose reduction or discontinuation (C)

Year	First author	ITPA Polymorphism	(A) Hemoglobin decline		(B) Severe anemia		(C) Ribavirin dose reduction or stop treatment	
			Cut-off Hb	Time of analysis	Cut-off Hb	Time of analysis	Cut-off Hb	Time of analysis
2010	Fellay [8]	rs1127354 rs7270101 Haplotype	>3 g/dL	4 weeks	<10 g/dL	4 weeks	NR	NR
2010	Thompson [10]	Haplotype	>3 g/dL	4 weeks	NR	NR	<8.5 g/dL	During treatment
2010	Sakamoto [15]	rs1127354	>3 g/dL	4 weeks	NR	NR	8.5–10 g/dL	4 weeks
2011	Thompson [16]	Haplotype	>3 g/dL	4/12/24 weeks	NR	NR	<9.5 g/dL	During treatment
2011	Azakami [17]	rs1127354	>2 g/dL	4 weeks	NR	NR	<8.5 g/dL	During treatment
2011	Chayama [36]	rs1127354	NR	NR	NR	NR	<12 g/dL	12 weeks
2011	Kurosaki [18]	rs1127354	>3 g/dL	4 weeks During treatment	<10 g/dL	4 weeks During treatment	NR	During treatment
2011	Rallón [19]	rs1127354 rs7270101 Haplotype	>2 g/dL	4 weeks	NR	NR	NR	NR
2012	Nishimura [20]	rs1127354	>3 g/dL	4 weeks	<10 g/dL	4 weeks	NR	NR
2012	Naggie [21]	Haplotype	>3 g/dL	4 weeks	NR	NR	NR	NR
2012	Domingo [22]	rs1127354	>3 g/dL	4 weeks	NR	NR	NR	During treatment
2012	Osinusi [23]	Haplotype	>3 g/dL	4 weeks	<10 g/dL	4 weeks	NR	NR
2012	Miyamura [37]	rs1127354	NR	NR	NR	NR	NR	4 weeks
2012	Vidal [33]	rs1127354	NR	NR	<10.5 g/dL	During treatment	NR	NR
2012	Tsubota [24]	rs1127354	>3 g/dL	4 weeks	<10 g/dL	4 weeks	NR	NR
2013	Rau [25]	rs1127354 rs7270101 Haplotype	>3 g/dL	During treatment	NR	NR	NR	NR
2013	Di Marco [26]	Haplotype	>3 g/dL	4 weeks	<10 g/dL	During treatment	NR	NR
2013	Ahmed [27]	rs1127354	>3 g/dL	4 weeks	<10 g/dL	12 weeks	NR	12 weeks
2013	Ogawa [34]	rs1127354	NR	NR	<8.5 g/dL	During treatment	NR	8–16 weeks
2013	Scherzer [28]	rs1127354 rs7270101 rs6051702	>3 g/dL	4 weeks	NR	NR	NR	NR
2013	Fujino [38]	rs1127354	NR	NR	NR	NR	NR	During treatment
2013	D’Avolio [29]	rs1127354 rs7270101 rs6051702	>3 g/dL	4 weeks	NR	NR	NR	NR
2013	Clark [30]	Haplotype	>3 g/dL	4 weeks	<10 g/dL	4 weeks	8.5–10 g/dL	During treatment
2013	Seto [39]	rs1127354	NR	NR	NR	NR	8.5–10 g/dL	During treatment
2013	Nakagawa [40]	rs1127354	NR	NR	NR	NR	NR	4 weeks
2014	Matsuura [31]	rs1127354	>3 g/dL	12 weeks	<10 g/dL	12 weeks	NR	NR
2014	Rembeck [41]	Haplotype	NR	NR	NR	NR	NR	During treatment
2014	Aghemo [35]	Haplotype	NR	NR	<8.9 g/dL	During treatment	8.5–10 g/dL	During treatment
2014	Hwang [32]	rs1127354 rs6051702	>3 g/dL	4 weeks	<10 g/dL NR	4 weeks NR	NR	NR

*Hb* hemoglobin, *NR* not reported

### Quality assessment

The quality scores of the studies included are summarized in Additional file 1: Table S1. The scores ranged from 9 to 18, with a mean value of 14 ± 1.98.

### Publication bias

The SNPs and haplotypes found in more than 10 articles were evaluated for publication bias tests. Analysis of publication bias was only necessary for rs1127354 in hemoglobin decline (Additional file 1: Figure S1A), severe anemia (Additional file 1: Figure S1B) and RBV dose reduction or discontinuation of treatment (Additional file 1: Figure S1C) meta-analyses. Nevertheless, the Egger’s test indicated that there was publication bias for rs1127354 (p = 0.019) in severe anemia meta-analysis.

### *ITPA* polymorphisms and hemoglobin decline

**Fig. 2:**
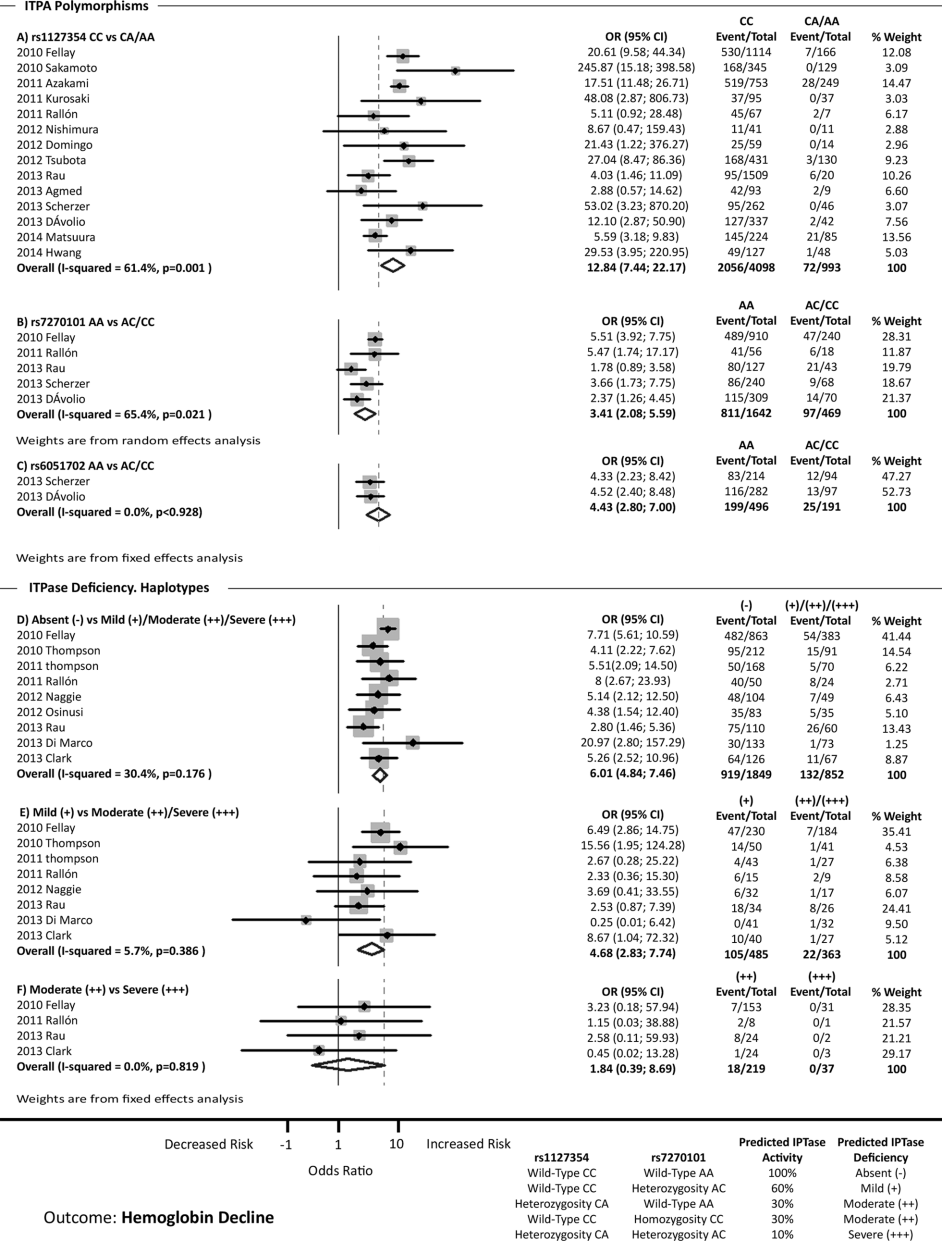
Forest plot shows the association between *ITPA* polymorphisms and hemoglobin decline. *CI* confidence intervals, *OR* odds ratio

A total of 20 references examined the association between *ITPA* polymorphisms and hemoglobin decline (Fig. 2).

The data for rs1127354 CC vs. CA/AA are shown in Fig. 2a. From 14 studies analyzed, 11 of them showed a significant association between rs1127354 CC genotype and hemoglobin decline. The pooled OR was 12.84 (95 % CI 7.44; 22.17), but a strong heterogeneity among the studies was found (I^2^ = 61.4 %; p = 0.001) (Fig. 2a). When the Galbraith plot was used, three outlier studies were identified: Sakamoto et al. [15], Azakami et al. [17], and Matsuura et al. [31] (Additional file 1: Figure S2A). A forest plot omitting these outliers was constructed, which reduced the heterogeneity (I^2^ = 39.9 %), but the pooled OR was not altered [OR = 12.35 (95 % CI 6.62; 23.04)]. The sensitivity analysis showed that no study should be excluded (Additional file 1: Figure S3A).

The data for rs7270101 AA vs. AC/CC are shown in Fig. 2b. From 5 studies analyzed, 4 of them showed a significant association between rs7270101 AA genotype and hemoglobin decline. The pooled OR was 3.41 (95 % CI 2.08; 5.59), but a strong heterogeneity among the studies was found (I^2^ = 65.4 %; p = 0.021) (Fig. 2b). When the Galbraith plot was used, one outlier of heterogeneity was identified: Fellay et al. [8] (Additional file 1: Figure S2B). When the mentioned outlier was deleted, a reduced heterogeneity was observed (I^2^ = 17.0 %), but the pooled OR was not altered [OR = 2.70 (95 % CI 1.78; 4.10)]. The sensitivity analysis showed that no study should be excluded (Additional file 1: Figure S3B).

The data for rs6051702 AA vs. AC/CC are shown in Additional file 1: Figure S4 and Fig. 2c. From 3 studies analyzed, 2 of them showed a significant association between rs6051702 AA genotype and hemoglobin decline. Nevertheless, the pooled OR was not significant [OR = 2.16 (95 % CI 0.54; 8.63)], but a strong heterogeneity was found among them (I^2^ = 92.4 %; p < 0.001) (Additional file 1: Figure S4). In this case, Galbraith´s plot identified one outlier as a source of heterogeneity: Hwang et al. [32] (Additional file 1: Figure S2C). Using sensitivity analysis (Additional file 1: Figure S3C) we found that the data from Hwang et al. [32] apparently influenced the overall results. Based on these issues, we considered the exclusion of this article from the analysis to be justified. When the meta-analysis was performed excluding this outlier, the heterogeneity disappeared (I^2^ = 0.0 %) and the pooled OR was significant [OR = 4.43 (95 % CI 2.80; 7.00)] (Fig. 2c). In any case, this analysis had a very small number of studies and the pooled OR should be considered with this taken into account.

*ITPA* haplotypes related to ITPase deficiency [8, 10] were also analysed.

The data for absent (−) vs. mild (+)/moderate (++)/severe (+++) ITPase deficiency are shown in Fig. 2d. All studies showed a significant association between absent (−) ITPase deficiency haplotype and hemoglobin decline. The pooled OR was 6.01 (95 % CI 4.84; 7.46). There was no significant heterogeneity among studies (I^2^ = 30.4 %; p = 0.176) (Fig. 2d). The sensitivity analysis showed that no study should be excluded (Additional file 1: Figure S3D).

The data for mild (+) vs. moderate (++)/severe (+++) ITPase deficiency are shown in Fig. 2e. From 8 studies analyzed, 3 of them showed a significant association between mild (+) ITPase deficiency haplotype and hemoglobin decline. The pooled OR was 4.68 (95 % CI 2.83; 7.74). There was no significant heterogeneity among studies (I^2^ = 5.7 %; p = 0.386) (Fig. 2e). The sensitivity analysis showed that no study should be excluded (Additional file 1: Figure S3E).

The data for moderate (++) vs. severe (+++) ITPase deficiency are shown in Fig. 2f. From 4 studies analyzed, none showed a significant association between moderate (++) ITPase deficiency and hemoglobin decline. The pooled OR was not significant [OR = 1.84 (95 % CI 0.39; 8.69)]. There was no significant heterogeneity among studies (I^2^ = 0.0 %; p = 0.819) (Fig. 2f). The sensitivity analysis showed that no study should be excluded (Additional file 1: Figure S3F).

### *ITPA* polymorphisms and severe anemia

**Fig. 3:**
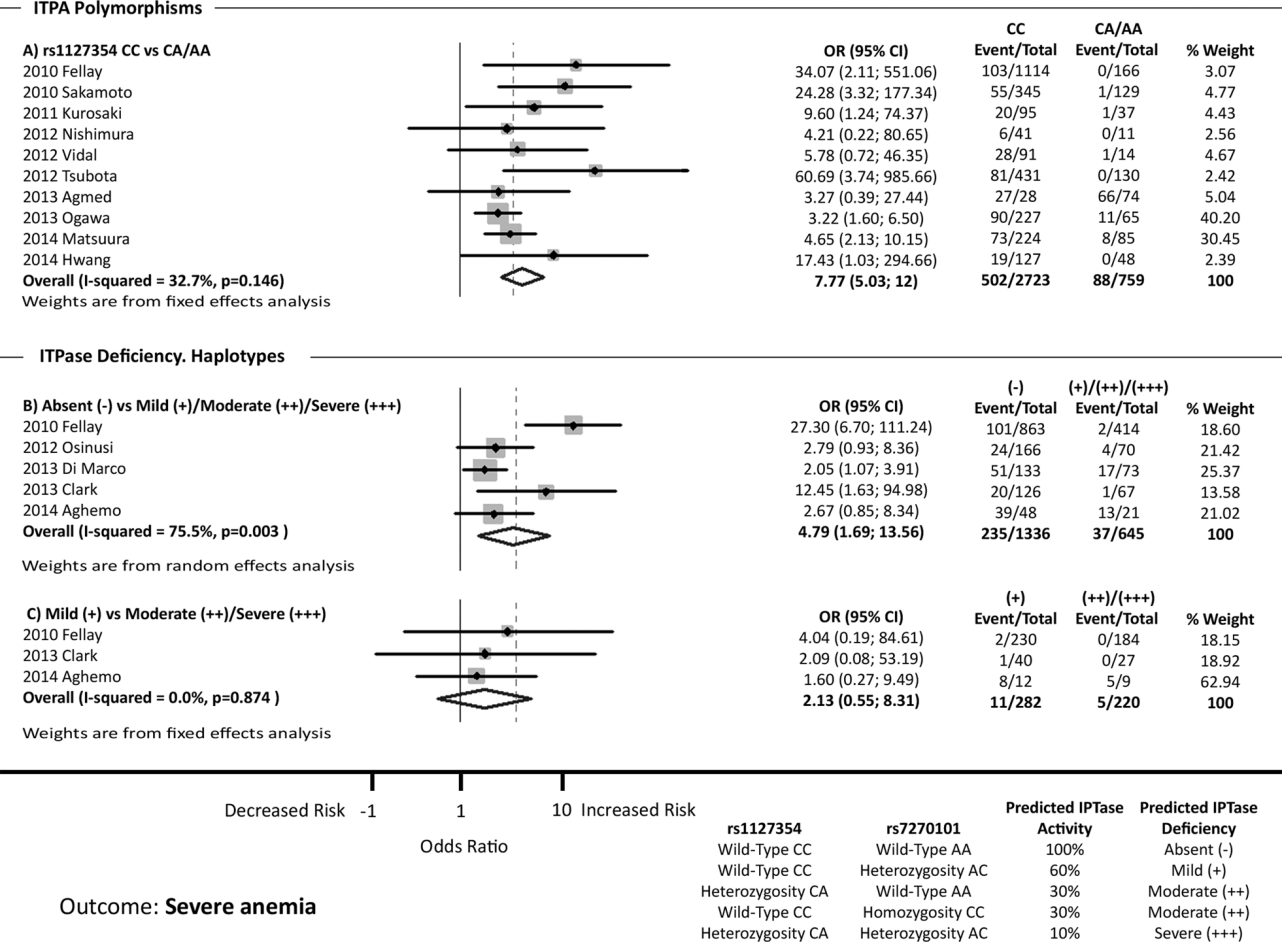
Forest plot shows the association between *ITPA* polymorphisms and severe anemia. *CI* confidence intervals, *OR* odds ratio

A total of 13 references were examined to evaluate the association between *ITPA* polymorphisms and severe anemia (Fig. 3). We were able to perform the analysis only for the *ITPA* rs1127354 polymorphism and two haplotypes that predict the deficiency of ITPase activity (absent and mild).

The data for rs1127354 CC vs. CA/AA are shown in Fig. 3a. From 10 studies analyzed, 7 of them showed a significant association between rs1127354 CC genotype and severe anemia. The pooled OR was 7.77 (95 % CI 5.03; 12.00). There was no significant heterogeneity among studies (I^2^ = 32.7 %; p = 0.146) (Fig. 3a). The sensitivity analysis showed that no study should be excluded (Additional file 1: Figure S5A).

The data for absent (−) vs. mild (+)/moderate (++)/severe (+++) ITPase deficiency are shown in Fig. 3b. From 5 studies analyzed, 3 showed a significant association between absent (−) ITPase deficiency haplotype and severe anemia. The pooled OR was 4.79 (95 % CI 1.69; 13.56), but a strong heterogeneity among the studies was found (I^2^ = 75.5 %; p = 0.003) (Fig. 3b). When the Galbraith plot was used, one outlier study was identified: Fellay et al. [8] (Additional file 1: Figure S2D). When the meta-analysis was performed excluding this outlier, the heterogeneity disappeared (I^2^ = 0.0 %), and the pooled OR decreased [OR = 2.80 (95 % CI 1.74; 4.51)]. The sensitivity analysis showed that no study should be excluded (Additional file 1: Figure S5B).

The data for mild (+) vs. moderate (++)/severe (+++) ITPase deficiency are shown in Fig. 3c. From 3 studies analyzed, none showed a significant association between mild (+) ITPase deficiency and severe anemia. Consequently, the pooled OR was not significant [OR = 2.13 (95 % CI 0.55; 8.31)]. There was no significant heterogeneity among studies (I^2^ = 0.0 %; p = 0.874) (Fig. 3c). The sensitivity analysis showed that no study should be excluded (Additional file 1: Figure S5C).

### *ITPA* polymorphisms and ribavirin-dose reduction or stop treatment

**Fig. 4:**
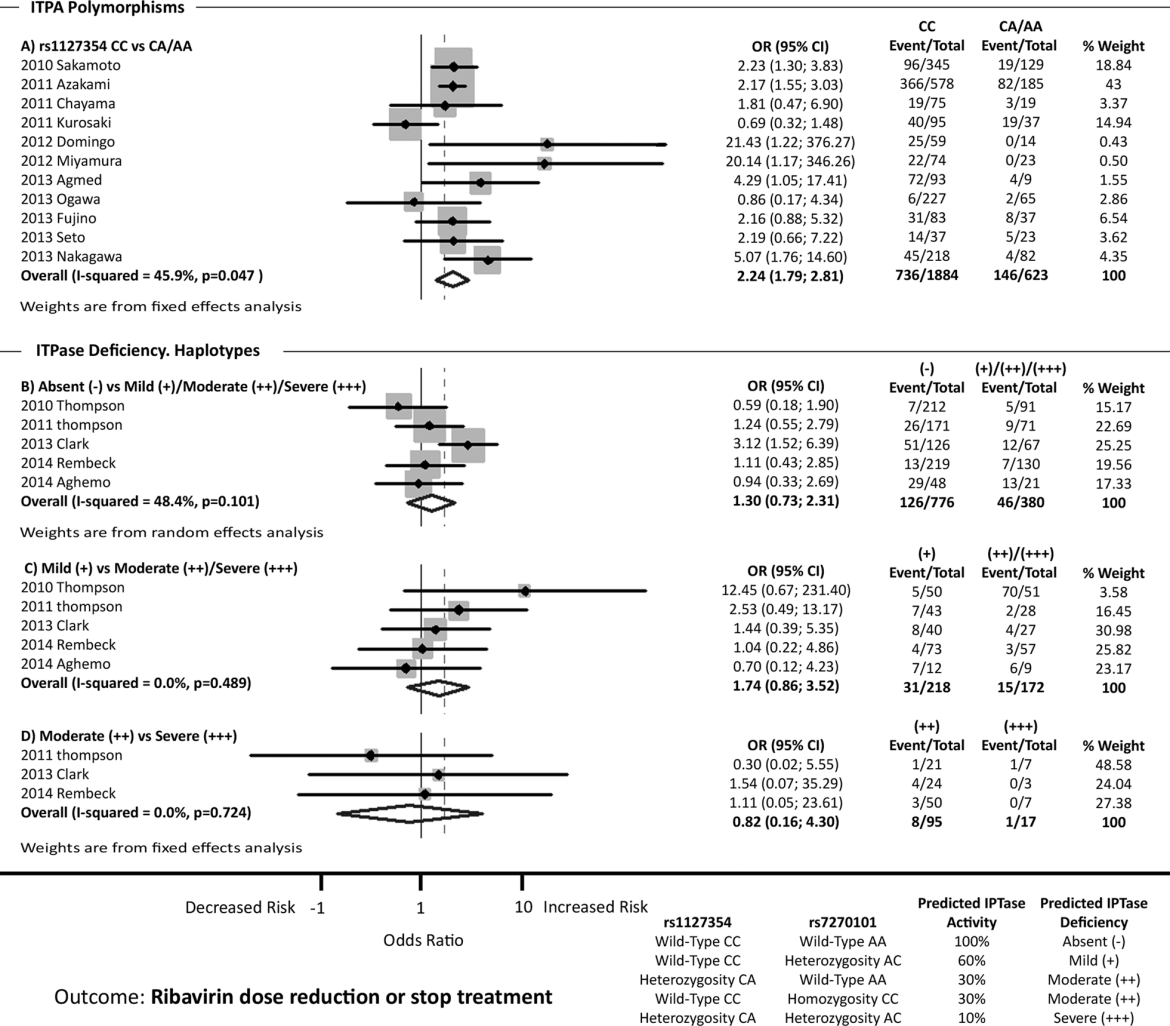
Forest plot shows the association between *ITPA* polymorphisms and ribavirin dose reduction or discontinuation. *CI* confidence intervals, *OR* odds ratio

A total of 16 references were examined to evaluate the association between *ITPA* polymorphisms and RBV dose reduction or discontinuation (Fig. 4). We were able to perform the analysis only for *ITPA* rs1127354 and the three haplotypes that predict the deficiency of ITPase activity.

The data for rs1127354 CC vs. CA/AA are shown in Fig. 4a. From 11 studies analyzed, 6 of them showed a significant association between rs1127354 CC genotype and RBV dose reduction or discontinuation. The pooled OR was 2.24 (95 % CI 1.79; 2.81), but heterogeneity was found among the studies (I^2^ = 45.9 %; p = 0.047) (Fig. 4a). When the Galbraith plot was used, one outlier study was identified: Kurosaki et al. [18] (Additional file 1: Figure S2E). A forest plot omitting the outlier was constructed, which reduced heterogeneity (I^2^ = 5.0 %; p = 0.394) and the pooled OR was not altered [OR = 2.52 (95 % CI 1.98; 3.20)]. Additionally, the sensitivity analysis showed that none of the 11 studies should be omitted from the analysis (Additional file 1: Figure S6A).

The data for absent (−) vs. mild (+)/moderate (++)/severe (+++) ITPase deficiency are shown in Fig. 4b. From 5 studies analyzed, only one showed a significant association between absent (−) ITPase deficiency haplotype and RBV dose reduction or discontinuation. The pooled OR was not significant [OR = 1.30 (95 % CI 0.73; 2.31)]. There was no significant heterogeneity among studies (I^2^ = 48.4 %; p = 0.101) (Fig. 4b). The sensitivity analysis showed that no study should be excluded (Additional file 1: Figure S6B).

The data for mild (+) vs. moderate (++)/severe (+++) ITPase deficiency are shown in Fig. 4c. From 5 studies analyzed, none showed a significant association between mild (+) ITPase deficiency and RBV dose reduction or discontinuation. Consequently, the pooled OR was not significant [OR = 1.74 (95 % CI 0.86; 3.52)]. There was no significant heterogeneity among studies (I^2^ = 0.0 %; p = 0.489) (Fig. 4c). The sensitivity analysis showed that no study should be excluded (Additional file 1: Figure S6C).

The data for moderate (++) vs. severe (+++) ITPase deficiency are shown in Fig. 4d. From 3 studies analyzed, none showed a significant association between moderate (++) ITPase deficiency and RBV dose reduction or discontinuation. Consequently, the pooled OR was not significant [OR = 0.82 (95 % CI 0.16; 4.30)]. There was not significant heterogeneity among studies (I^2^ = 0.0 %; p = 0.724) (Fig. 4d). The sensitivity analysis showed that no study should be excluded (Additional file 1: Figure S6D).

### Meta-regression analysis

Several factors were analyzed to investigate the possible influence on the heterogeneity (Additional file 1: Table S2). However, no significant association between these factors and the outcome variables was found (Additional file 1: Table S2).

## Discussion

The three major results of our meta-analysis were: (1) The presence of the major alleles in homozygosis for *ITPA* polymorphisms (rs1127354 CC, rs7270101 AA, and rs6051702 AA) was associated with a higher chance of developing hemoglobin decline. Additionally, significant associations with a higher chance of developing hemoglobin decline were found both absent (−) and mild (+) ITPase deficiency haplotypes. (2) The *ITPA* rs1127354 polymorphism and absent (−) ITPase deficiency haplotype were associated with severe anemia. (3) The rs1127354 CC genotype showed a significant association with RBV dose reduction or discontinuation of treatment.

Hemolytic anemia is an important side-effect in RBV-based HCV therapy [2–4]. The toxicity is RBV concentration-dependent and anemia improves upon dose reduction [5], but high variability limits the prediction of anemia based on RBV plasma concentrations. Clinical risk factors for severe RBV-induced anemia include impaired renal function, age, dose per body weight, female gender, baseline platelet levels, baseline hemoglobin levels, and haptoglobin phenotype [42–44]. In this context, the identification of successful predictors of RBV-induced anemia is of great value for preventing its toxicity.

In our meta-analysis, we found a significant association between the unfavorable *ITPA* genotypes (homozygous major alleles) of the three SNPs studied (rs1127354, rs7270101 and rs6051702) and RBV-induced hemolytic anemia. In most of the studies, the odds of developing anemia was more than double in patients with an unfavorable *ITPA* genotype than in patients with a protective *ITPA* minor variant. However, the magnitude of the association was different in many cases. This may be due to the different criteria considered in each individual study.

Although *ITPA* polymorphisms have been associated with hemoglobin decline, few studies have compared the predictive value of combining the three *ITPA* polymorphisms, probably because of the scattered distribution of these *ITPA* polymorphisms among diverse ethnic groups and world populations. In our meta-analysis, a simultaneous evaluation of the three *ITPA* SNPs (rs1127354, rs7270101 and rs6051702) was done when possible, but the rs6051702 polymorphism was rarely encountered and often there was too little data to draw robust conclusions on all three. The *ITPA* rs1127354 polymorphism (P32T substitution) exists at low frequency in Central and South American populations (1–2 %), at a constant frequency across Caucasian and African populations (6–7 %), and at the highest frequencies in Asian populations (14–19 %) [45]. Also, the rs7270101 is known to not be polymorphic in the Japanese population [46]. For these reasons, race was analyzed in the meta-regression as a covariate, but it was not a determinant of heterogeneity in the outcomes analyzed. However, we think that this is probably due to the preselection of *ITPA* polymorphisms according to the study population.

The decrease in hemoglobin levels was the outcome most frequently studied and the only one that provided enough data to compare the three *ITPA* polymorphisms. There were two studies that showed the three SNPs at the same time [28, 29], and our meta-analysis was in agreement with them. The rs1127354 polymorphism was associated with higher odds of hemoglobin reduction, whereas rs7270101 and rs6051702 also showed associations in the same direction albeit to a lesser degree. The haplotypes related to a higher predicted ITPase activity, defined by the presence of *ITPA* major alleles at the polymorphic sites rs1127354 and rs7270101, also showed a similar trend. Relationships in the same direction were found for severe anemia, but with fewer studies. However, almost no significant associations were found for *ITPA* polymorphisms or haplotypes with RBV dose reduction or discontinuation. This might be explained by the multiple factors influencing this outcome or due to the inclusion of different events in the same endpoint (RBV dose reduction and/or discontinuation), which are reported at different times during treatment. In addition, the need for therapy modification may be influenced by other clinical factors that were not taken into account. Of special interest is the use of erythropoietin (EPO) to improve hemoglobin levels and limit the need to reduce the dose of RBV [47]. To date, no prospective trials have been performed to definitely demonstrate that the use of EPO has a positive impact on SVR, but EPO is administered in some countries when the hemoglobin level falls below 10 g/dL or by physician criteria. [2, 3]. Despite this, no general consensus exists regarding the use of EPO, and it is not available to treat this condition everywhere. This meta-analysis includes seven studies in which the use of EPO was allowed [19, 22, 23, 26, 30, 33, 35], but erythropoietin was not considered as a covariate because: (1) the percentage of patients who received erythropoietin was low (3 %) (data not shown); (2) some studies did not provide this information or is not detailed; and (3) in most cases, the anemia occurred before treatment with erythropoietin was administered.

The heterogeneity of the studies must be taken into account in a meta-analysis. The covariates from the study population that were included in the analysis were age, gender and HIV coinfection, which have been previously described as risk factors for RBV-induced anemia [19, 42]. Another cause of heterogeneity is the definitions of the clinical endpoints established in each study. Fellay et al. [8] were the pioneers in these studies, and the clinical endpoints that they established have been followed by other authors. The hemoglobin reduction of 3 g/dL or more within 4 weeks of starting treatment is considered to be a significant indicator of anemia. The hemoglobin value <10 g/dL is the level at which RBV dose reduction is recommended, and when hemoglobin is <8.5 g/dL, RBV therapy is stopped permanently, according to international guidelines [2, 3]. However, other authors have categorized anemia as mild, moderate or severe according to a modification of the World Health Organization scale (International Statistical Classification of Diseases and Related Health Problems) [33]. The time of analysis is also heterogeneous, since clinical endpoints have been reported at 4 weeks, 12 weeks or any time during treatment. The time on HCV therapy is an important factor in the development of anemia. Moreover, the HCV genotype affects the type and duration of treatment since patients with GT2/3 are prescribed 24-week treatments and patients with GT1/4 undergo treatments of 48 weeks. Besides, the administered RBV doses are adjusted according to HCV genotype and responsiveness to treatment. For these reasons, we analyzed these factors in our meta-analysis, finding that they did not constitute a significant source of heterogeneity.

The severity of RBV-related anemia is known for long but it is difficult to assess the role of each drug (RBV, pegIFNα or DAAs) in the anemia. Although most of the studies have been performed in patients on combined therapy (pegIFNα/RBV), a strong association between an *ITPA* SNP and anemia has been reported in patients on RBV monotherapy [48]. Moreover, the inclusion of DAAs in HCV therapy should be taken into consideration when studying anemia in HCV-infected patients, since the first generation of protease inhibitors (boceprevir and telaprevir) has been found to be associated with an increased anemia frequency and severity [49]. Data from telaprevir-based triple therapy patients are provided by three of the studies included in our meta-analysis [34–36], with a frequency of severe anemia over 75 %. However, some discrepancies have been detected regarding these data. While some authors reported that *ITPA* rs1127354 is a useful predictor of the development of severe anemia in telaprevir-based triple therapy patients [34, 36], Aghemo et al. reported that an *ITPA* polymorphism was not associated with early anemia or therapy modification [35]. These differences may be due to SNPs studied, the selected population (all in advanced stages of fibrosis) and the limited of sample size. In any case, although no significant results were found, the same trend was observed for telaprevir-based therapy as in the overall results.

To date, no data have been available about *ITPA* polymorphisms and the second-generation of DAAs, such as sofosbuvir, simeprevir, and daclatasvir. Note that patients undergoing triple therapy with pegIFN/RBV plus these new DAAs have similar anemia frequencies and profiles as patients receiving pegIFN/RBV alone [50, 51]. In a recent study, 72 % of patients who received simeprevir or sofosbuvir plus RBV developed anemia requiring intervention [52]. Thus, *ITPA* polymorphisms will still be useful in preventing anemia while RBV continues to be included in HCV treatment regimens.

Finally, in order to properly interpret our results, some considerations have to be taken into account. Firstly, our meta-analysis was performed by using the unadjusted raw data provided from each study, whereas most of the results given by the authors had been adjusted by age, gender, HCV viral load, and/or other factors. For this reason, the pooled ORs may differ slightly from those reported in the original articles. Secondly, renal function, baseline hemoglobin level and other variables involved in determining individual susceptibility to RBV-induced anemia have not been considered. Thirdly, most studies reported the results as haplotype, which does not allow the results of each polymorphism to be evaluated separately. Fourthly, the majority of the studies had a retrospective design and the number of studies in some subgroup analyses was small, which might have led to weak results. Consequently, these results should be interpreted with caution.

In conclusion, *ITPA* polymorphisms increase the likelihood of developing hemolytic anemia in HCV-infected patients on RBV-based therapy, rs1127354 CC and rs7270101 AA genotypes, suggesting the utility of screening for *ITPA* polymorphisms as a way to avoid hematological toxicity and increase adherence to RBV-based therapy.

## Additional file

10.1186/s12967-015-0682-y Methodological quality of the studies included in the meta-analysis, which was assessed using a modified score based in the Newcastle–Ottawa Scale (Stang A. Critical evaluation of the Newcastle–Ottawa scale for the assessment of the quality of nonrandomized studies in meta-analyses. Eur J Epidemiol 2010;25:603–605). (see Supplemental Box 2). **Table S2.** Meta-regression analysis for hemoglobin decline (A–C), severe anemia (D) and ribavirin dose reduction or discontinuation (E) according to ITPA polymorphisms. **Box 1.** Description of rs1127354/rs7270101 haplotype: ITPase deficiency ranged from absent (-) (representing wild-type activity) to mild (+), moderate (++) or severe (+++) (8, 10). **Box 2.** Methodological criteria used to evaluate the quality of the studies included in the meta-analysis. This score was adapted from Newcastle–Ottawa Scale (Stang A. Critical evaluation of the Newcastle-Ottawa scale for the assessment of the quality of nonrandomized studies in meta-analyses. Eur J Epidemiol 2010;25:603–605). **Figure S1.** Publication bias for the rs1127354 studies included in the meta-analysis for hemoglobin decline(A), severe anemia (B) and ribavirin dose reduction or discontinuation (C) according both to Begg’s funnel plots and Egger’s test. Abbreviations: Coef., asymmetry regression coefficient; Std.Err., standard error; t, statistic; P>|t|, significance; and CI, confidence interval. Coefficients correspond to the intercept value in the regression equation, which estimates the asymmetry of the funnel plot. Positive values (Coef.> 0) indicate higher levels of effect size in studies with smaller sample sizes. **Figure S2.** Publication heterogeneity for studies included in the meta-analysis for hemoglobin decline: rs1127354 (A), rs7270101 (B), rs6051702 (C); severe anemia: haplotype rs1127354CC / rs7270101AA (D); and ribavirin dose reduction or discontinuation: rs1127354 (E); according to Galbraith’s plots. Abbreviations 1/s.e, precision; b/s.e, standardized effect. **Figure S3.** Sensitivity analysis for studies included in the meta-analysis for hemoglobin decline: rs1127354 (A), rs7270101 (B), rs6051702 (C), haplotype absent (-) vs. mild (+)/moderate (++)/severe (+++) (D), haplotype mild (+) vs. moderate (++)/severe (+++) (E) and moderate (++) vs. severe (+++) (F). Sensitivity analyses were carried out to investigate the influence of any one study on the overall meta-analysis by sequential omission of individual studies. **Figure S4.** Forest plot of the meta-analysis performed to investigate the association between ITPA rs6051702 polymorphisms and hemoglobin decline, included in the Hwang, et al. Article (32). Abbreviations: CI, confidence intervals; OR, odds ratio. **Figure S5.** Sensitivity analysis for studies included in the meta-analysis for severe anemia: rs1127354 (A), haplotype absent (-) vs. mild (+)/moderate (++)/severe (+++) (B) and haplotype mild (+) vs. moderate (++)/severe (+++) (C). Sensitivity analyses were carried out to investigate the influence of any one study on the overall meta-analysis by sequential omission of individual studies. **Figure S6.** Sensitivity analysis for studies included in the meta-analysis for ribavirin dose reduction or discontinuation: rs1127354 (A), haplotype absent (-) vs. mild (+)/moderate (++)/severe (+++) (B), haplotype mild (+) vs. moderate (++)/severe (+++) (C) and moderate (++) vs. severe (+++) (D). Sensitivity analyses were carried out to investigate the influence of any one study on the overall meta-analysis by sequential omission of individual studies.

## Abbreviations

95 % CI: 95 % of confidence interval
ATP: adenosin triphosphate
DAAs: direct-acting antivirals
GT: HCV genotype
HCV: hepatitis C virus
HIV: human immunodeficiency virus
ITP: inosine triphosphate
ITPA: inosine triphosphatase
ITPase: inosine triphosphate pyrophosphohydrolase
OR: odds ratio
pegIFNα: pegylated interferon-alpha
RBV: ribavirin
SNP: single nucleotide polymorphism
