# Supplementary material for: Relationship between ITPA polymorphisms and hemolytic anemia in HCV-infected patients after ribavirin-based therapy: a meta-analysis
Source: J Transl Med. 2015 Oct 6;13:320. doi: 10.1186/s12967-015-0682-y (PMC4595047; doi:10.1186/s12967-015-0682-y)

**Supplemental Table 1.** Methodological quality of the studies included in the meta-analysis, which was assessed using a modified score based in the Newcastle–Ottawa Scale (*Stang A. Critical evaluation of the Newcastle-Ottawa scale for the assessment of the quality of nonrandomized studies in meta-analyses. Eur J Epidemiol 2010;25:603-605*). (see Supplemental Box 2).

| **Year** | **First author** | **SELECTION** | | | | **ANALYSIS** | | | | | | **RESULTS** | | | **Total Score** |
| --- | --- | --- | --- | --- | --- | --- | --- | --- | --- | --- | --- | --- | --- | --- | --- |
|  |  | 1 | 2 | 3 | **Subtotal** | 4 | 5 | 6 | 7 | 8 | **Subtotal** | 9 | 10 | **Subtotal** |  |
| **2010** | **Fellay** | ** | ** | * | 5 | ** | ** | ** | ** | ** | 10 | ** | * | 3 | 18 |
| **2010** | **Thompson** | ** | ** | ** | 6 | ** | ** | ** | - | ** | 8 | ** | ** | 4 | 18 |
| **2010** | **Sakamoto** | ** | ** | * | 5 | ** | ** | - | - | ** | 6 | ** | * | 3 | 14 |
| **2011** | **Thompson** | ** | ** | ** | 6 | ** | ** | - | - | ** | 6 | * | ** | 3 | 15 |
| **2011** | **Azakami** | ** | ** | ** | 6 | ** | ** | - | - | * | 5 | * | ** | 3 | 14 |
| **2011** | **Chayama** | ** | ** | ** | 6 | ** | - | - | - | * | 3 | ** | ** | 4 | 13 |
| **2011** | **Kurosaki** | ** | ** | ** | 6 | ** | ** | - | - | ** | 6 | ** | ** | 4 | 16 |
| **2011** | **Rallón** | ** | * | * | 4 | ** | - | ** | - | ** | 6 | ** | * | 3 | 13 |
| **2012** | **Nishimura** | ** | * | * | 4 | ** | - | * | - | * | 4 | ** | ** | 4 | 12 |
| **2012** | **Naggie** | ** | ** | * | 5 | ** | * | ** | - | ** | 7 | - | - | 0 | 12 |
| **2012** | **Domingo** | ** | ** | ** | 6 | ** | - | - | - | ** | 4 | * | * | 2 | 12 |
| **2012** | **Osinusi** | ** | ** | * | 5 | ** | * | - | - | ** | 5 | ** | * | 3 | 13 |
| **2012** | **Miyamura** | ** | ** | - | 4 | - | - | - | - | * | 1 | ** | ** | 4 | 9 |
| **2012** | **Vidal** | ** | ** | * | 5 | ** | * | ** | - | ** | 7 | ** | - | 2 | 14 |
| **2012** | **Tsubota** | ** | ** | ** | 6 | ** | ** | - | - | ** | 6 | ** | ** | 4 | 16 |
| **2013** | **Rau** | * | ** | ** | 5 | * | * | - | - | ** | 4 | ** | ** | 4 | 13 |
| **2013** | **Di Marco** | ** | * | ** | 5 | ** | ** | - | - | ** | 6 | ** | ** | 4 | 15 |
| **2013** | **Ahmed** | ** | ** | ** | 6 | ** | * | ** | - | ** | 7 | ** | * | 3 | 16 |
| **2013** | **Ogawa** | ** | ** | ** | 6 | ** | ** | - | - | * | 5 | ** | ** | 4 | 15 |
| **2013** | **Scherzer** | ** | ** | * | 5 | ** | ** | - | - | * | 5 | ** | ** | 4 | 14 |
| **2013** | **Fujino** | ** | ** | ** | 6 | ** | * | - | - | * | 4 | ** | * | 3 | 13 |
| **2013** | **D'Avolio** | ** | * | ** | 5 | ** | ** | ** | - | ** | 8 | ** | ** | 4 | 17 |
| **2013** | **Clark** | * | ** | ** | 5 | ** | * | ** | - | * | 6 | ** | ** | 4 | 15 |
| **2013** | **Seto** | ** | ** | * | 5 | ** | ** | - | - | ** | 6 | ** | ** | 4 | 15 |
| **2013** | **Nakagawa** | * | ** | ** | 5 | * | - | ** | - | * | 4 | ** | * | 3 | 12 |
| **2014** | **Matsuura** | ** | ** | * | 5 | ** | ** | ** | - | * | 7 | ** | * | 3 | 15 |
| **2014** | **Rembeck** | * | ** | ** | 5 | ** | - | - | - | * | 3 | ** | * | 3 | 11 |
| **2014** | **Aghemo** | ** | ** | ** | 6 | ** | ** | - | - | ** | 6 | ** | * | 3 | 15 |
| **2014** | **Hwang** | ** | ** | * | 5 | ** | * | - | - | ** | 5 | ** | * | 3 | 13 |
| **Quality average ± standard deviation:** | | | | | **5±0.63** |  |  |  |  |  | **5±0.76** |  |  | **3±0.87** | **14±1.98** |
| Maximum score: | | | | | 6 |  |  |  |  |  | 10 |  |  | 4 | 20 |

**Supplemental Table 2.** Meta-regression analysis for hemoglobin decline (A-C), severe anemia (D) and ribavirin dose reduction or discontinuation (E) according to *ITPA* polymorphisms.

| **Outcome** | **Coefficient** | **Standard error** | **P-value** | **95% CI** |
| --- | --- | --- | --- | --- |
|  | | | | |
| **Hemoglobin decline** | | | | |
| **A) rs1127354 CC vs CA/AA** |  |  |  |  |
| **Cut-off** | 0.292 | 0.3772 | 0.453 | -0.523; 1.107 |
| **Time of analysis** | -0.226 | 0.256 | 0.393 | -0.779; 0.326 |
| **Sex** | -0.143 | 0.168 | 0.411 | -0.506; 0.221 |
| **Age** | 0.239 | 0.188 | 0.901 | -0.387; 0.435 |
| **Race** | 0.313 | 0.283 | 0.288 | -0.298; 0.925 |
| **HCV genotype** | -0.136 | 0.541 | 0.805 | -1.305; 1.032 |
| **HCV therapy** | - | - | - | - |
| **HIV coinfection** | -0.385 | 0.980 | 0.701 | -2.503; 1.733 |
| **B) rs7270101 AA vs AC/CC** |  |  |  |  |
| **Cut-off** | 0.583 | 0.705 | 0.469 | -1.661; 2.828 |
| **Time of analysis** | -0.754 | 0.431 | 0.178 | -2.125; 0.617 |
| **Sex** | 0.296 | 0.326 | 0.432 | -0.743; 1.334 |
| **Age** | -0.181 | 0.466 | 0.735 | -2.188; 1.824 |
| **Race** | 0.510 | 0.296 | 0.184 | -0.435; 1.455 |
| **HCV genotype** | -0.587 | 0.271 | 0.119 | -1.449; 0.275 |
| **HCV therapy** | - | - | - | - |
| **HIV coinfection** | 0.583 | 0.705 | 0.469 | -1.661; 2.828 |
| **C) rs6051702 AA vs AC/CC** |  |  |  |  |
| **Cut-off** | 1.047 | 1.896 | 0.679 | -23.049; 25.144 |
| **Time of analysis** | -0.769 | 0.286 | 0.227 | -4.407; 2.868 |
| **Sex** | - | - | - | - |
| **Age** | - | - | - | - |
| **Race** | -1.085 | 0.212 | 0.123 | -3.787; 1.618 |
| **HCV-Genotype** | -1.048 | 1.896 | 0.679 | -25.145; 23.049 |
| **HCV therapy** | - | - | - | - |
| **HIV coinfection** | - | - | - | - |
|  |  |  |  |  |
| **Severe anemia** | | | | |
| **D) Absent (-) vs Mild (+)/Moderate (++)/Severe (+++)** | | | | |
| **Cut-off** | -0.171 | 0.401 | 0.687 | -1.204; 0.860 |
| **Time of analysis** | -0.969 | 0.773 | 0.265 | -2.956; 1.016 |
| **Sex** | -0.516 | 0.407 | 0.261 | -1.563; 0.530 |
| **Age** | -0.307 | 0.719 | 0.698 | -2.595; 1.980 |
| **Race** | 0.878 | 1.355 | 0.563 | -3.434; 5.191 |
| **HCV genotype** | -0.992 | 0.848 | 0.295 | -3.172; 1.188 |
| **HCV therapy** | -0.515 | 1.205 | 0.678 | -3.612; 2.581 |
| **HIV coinfection** | -0.422 | 0.616 | 0.524 | -2.007; 1.162 |
|  |  |  |  |  |
| **Ribavirin dose reduction or stop treatment** | | | | |
| **E) rs1127354 CC vs CA/AA** | | | | |
| **Cut-off** | 0.013 | 0.162 | 0.937 | -0.354; 0.381 |
| **Time of analysis** | -0.309 | 0.286 | 0.309 | -0.955; 0.338 |
| **Sex** | 0.188 | 0.150 | 0.241 | -0.151; 0.527 |
| **Age** | -0.150 | 0.169 | 0.398 | -0.539; 0.238 |
| **Race** | -0.566 | 0.394 | 0.185 | -1.456; 0.325 |
| **HCV genotype** | 0.574 | 0.305 | 0.092 | -0.116; 1.264 |
| **HCV therapy** | -0.580 | 0.711 | 0.436 | -2.187; 1.028 |
| **HIV coinfection** | 2.337 | 1.620 | 0.183 | -1.326; 6.002 |

**Abbreviations:** CI, confidence interval; HCV, hepatitis C virus; HIV, human inmunodefficiency virus

**Supplemental Box 1.** Description of rs1127354/rs7270101 haplotype: ITPase deficiency ranged from absent (-) (representing wild-type activity) to mild (+), moderate (++) or severe (+++) .

| **rs1127354** | **rs7270101** | **Predicted ITPase Activity** | **Predicted ITPase Deficiency** |
| --- | --- | --- | --- |
| Wild-Type CC | Wild-Type AA | 100% | Absent (-) |
| Wild-Type CC | Heterozygosity AC | 60% | Mild (+) |
| Heterozygosity CA | Wild-Type AA | 30% | Moderate (++) |
| Wild-Type CC | Homozygosity CC | 30% | Moderate (++) |
| Heterozygosity CA | Heterozygosity AC | 10% | Severe (+++) |

**Supplemental Box 2.** Methodological criteria used to evaluate the quality of the studies included in the meta-analysis. This score was adapted from Newcastle–Ottawa Scale (*Stang A. Critical evaluation of the Newcastle-Ottawa scale for the assessment of the quality of nonrandomized studies in meta-analyses. Eur J Epidemiol 2010;25:603-605*).

| **Methodological quality criteria** |
| --- |
| **SELECTION** |
| **1.- Adequate definition of populations:**  A. Complete description (age, sex, viral load and viral genotype): **  B. Partial description: *  C. No description: - |
| **2.- Adequate definition of treatment:**  A. Complete description (therapy, doses and duration): **  B. Partial description: *  C. No description: - |
| **3.- Adequate definition of inclusion/exclusion criteria:**  A. Complete description (both criteria): **  B. Partial description (both criteria) or complete description of one of them: *  C. No description: - |
| **ANALYSIS** |
| **4.- Adequate statistical approach:**  A. Adjusted regression analysis: **  B. Univariate regression analysis: *  C. No regression: - |
| **5.- Statistical power:**  A. n > 200: **  B. 100 ≤ n ≤ 200: *  C. n<100: - |
| **6.- Hardy-Weinberg equilibrium:**  A. Yes (p > 0.05): **  B. No (p < 0.05): *  C. No evaluated: - |
| **7.- Adequate definition of genetic model of inheritance:**  A. Description of the genetic model and the selection criteria: **  B. Description of the genetic model: *  C. No description: - |
| **8.- Definition of clinical endpoints**  A. Complete description (time and cut-off): **  B. Partial description: *  C. No description: - |
| **RESULTS** |
| **9.- Outcomes measurement well described and without incongruences**  A. No incongruences: **  B. Minor incongruences: *  C. Major incongruences: - |
| **10.- Raw data given or calculable**  A. Data provided: **  B. Estimated from crude data: *  C. Estimated indirectly: - |

**Supplemental Figure 1.** Publication bias for the rs1127354 studies included in the meta-analysis for hemoglobin decline (A), severe anemia (B) and ribavirin dose reduction or discontinuation (C) according both to Begg’s funnel plots and Egger’s test. **Abbreviations:** Coef., asymmetry regression coefficient; Std.Err., standard error; t, statistic; P>|t|, significance; and CI, confidence interval.

Coefficients correspond to the intercept value in the regression equation, which estimates the asymmetry of the funnel plot. Positive values (Coef.> 0) indicate higher levels of effect size in studies with smaller sample sizes.

**
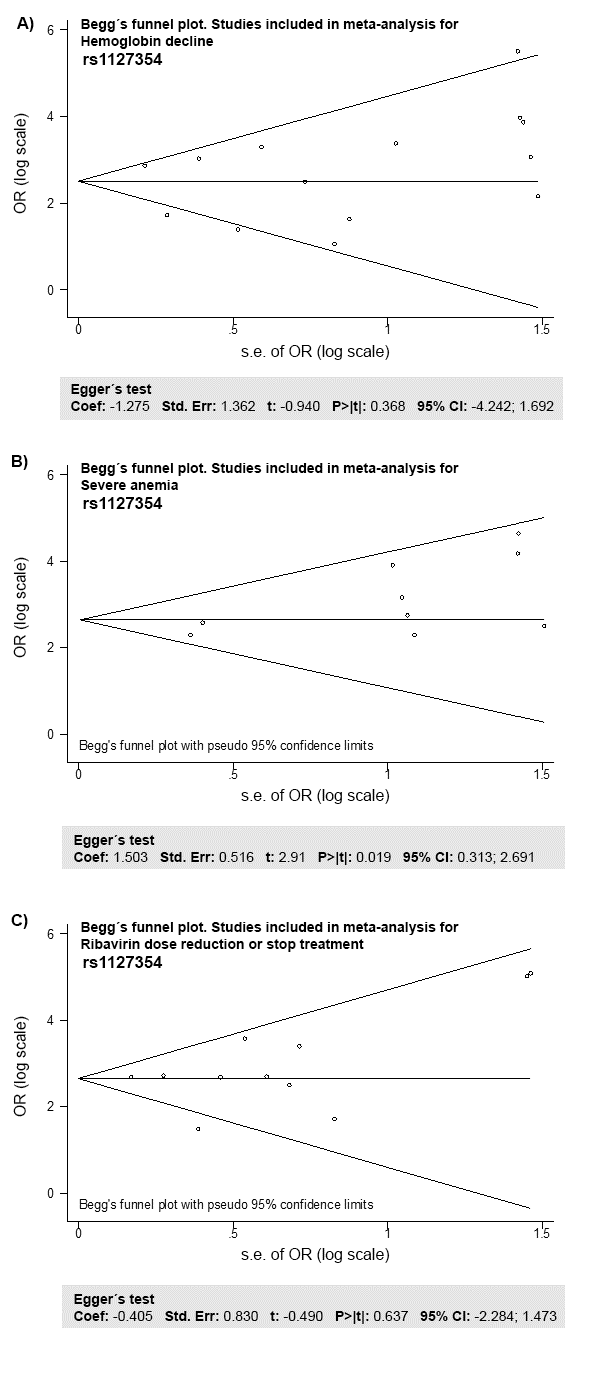
**

**Supplemental Figure 2.** Publication heterogeneity for studies included in the meta-analysis for hemoglobin decline: rs1127354 (A), rs7270101 (B), rs6051702 (C); severe anemia: haplotype rs1127354CC / rs7270101AA (D); and ribavirin dose reduction or discontinuation: rs1127354 (E); according to Galbraith’s plots. **Abbreviations** 1/s.e, precision; b/s.e, standardized effect.


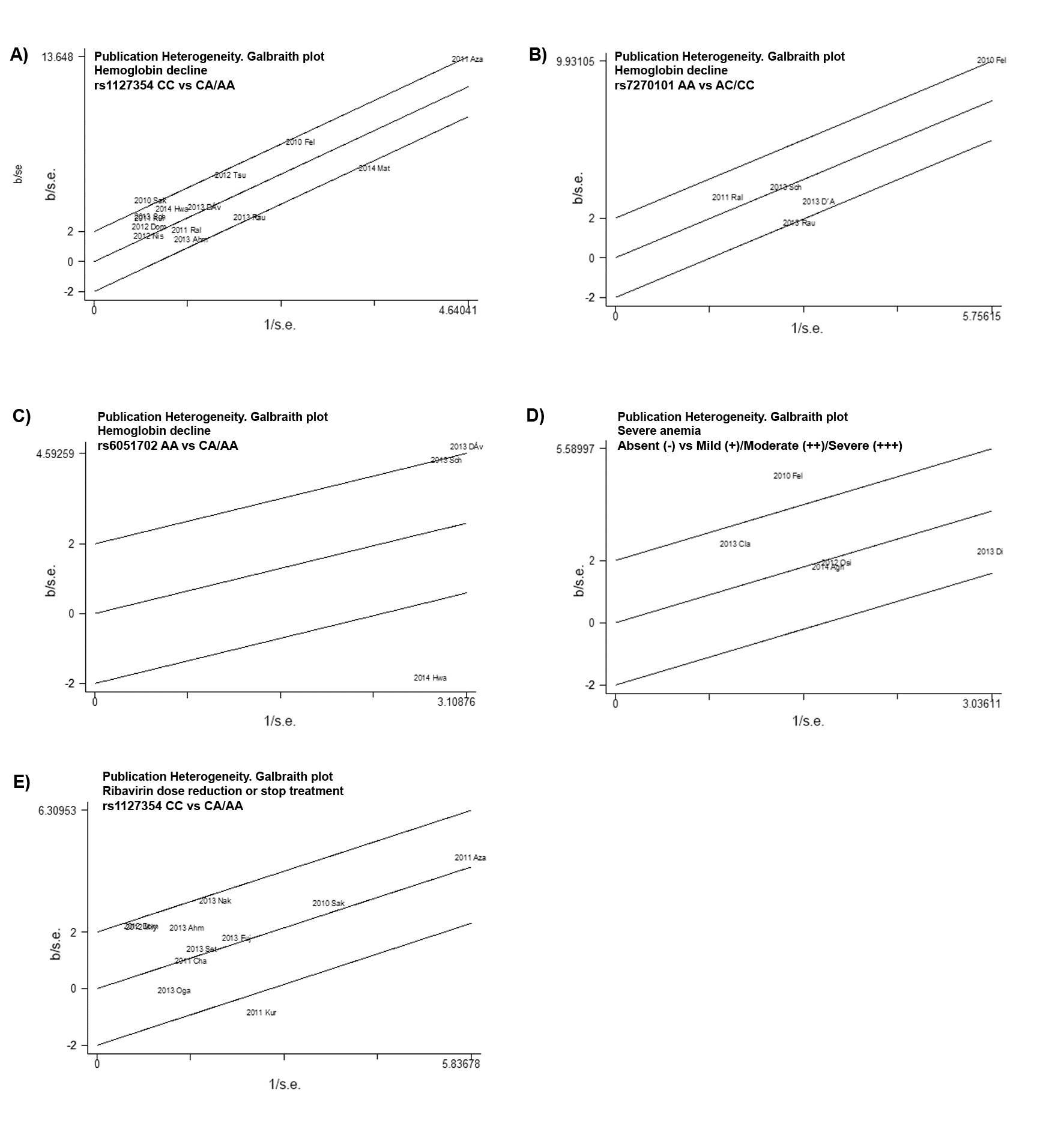


**Supplemental Figure 3.** Sensitivity analysis for studies included in the meta-analysis for hemoglobin decline: rs1127354 (A), rs7270101 (B), rs6051702 (C), haplotype absent (-) vs. mild (+)/moderate (++)/severe (+++) (D), haplotype mild (+) vs. moderate (++)/severe (+++) (E) and moderate (++) vs. severe (+++) (F). Sensitivity analyses were carried out to investigate the influence of any one study on the overall meta-analysis by sequential omission of individual studies.

**
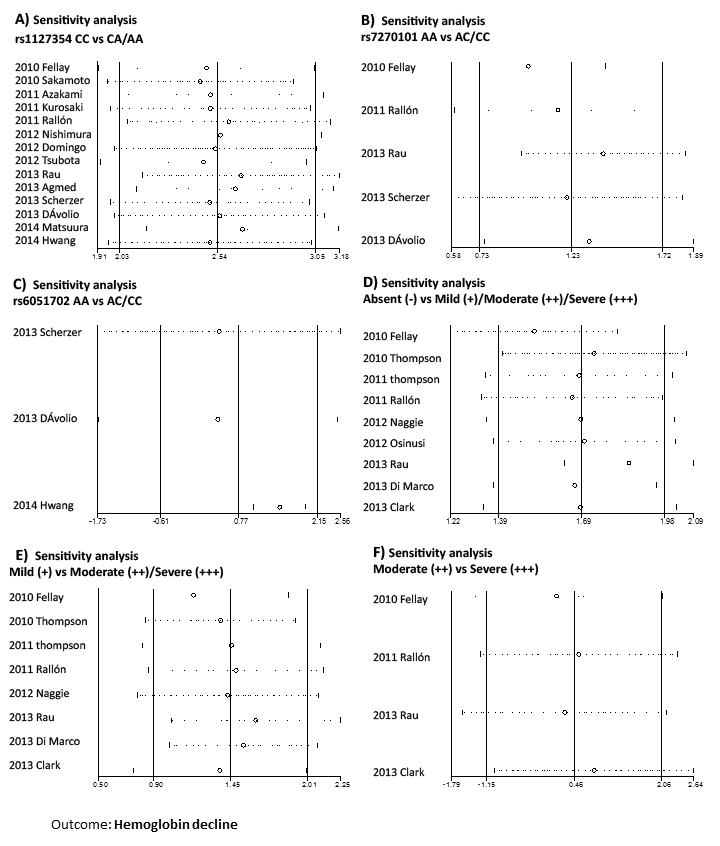
**

**Supplemental Figure 4.** Forest plot of the meta-analysis performed to investigate the association between *ITPA* rs6051702 polymorphisms and hemoglobin decline, included in the Hwang, et al. article .

**Abbreviations:** CI, confidence intervals; OR, odds ratio.


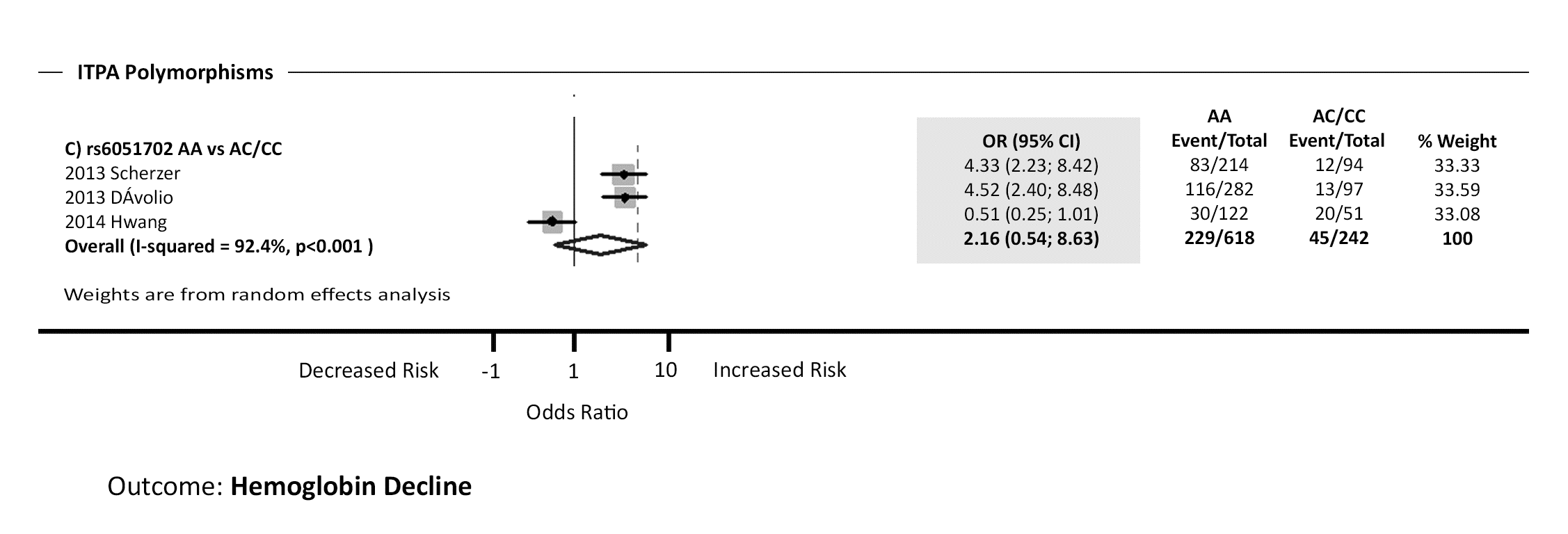


**Supplemental Figure 5.** Sensitivity analysis for studies included in the meta-analysis for severe anemia: rs1127354 (A), haplotype absent (-) vs. mild (+)/moderate (++)/severe (+++) (B) and haplotype mild (+) vs. moderate (++)/severe (+++) (C). Sensitivity analyses were carried out to investigate the influence of any one study on the overall meta-analysis by sequential omission of individual studies.


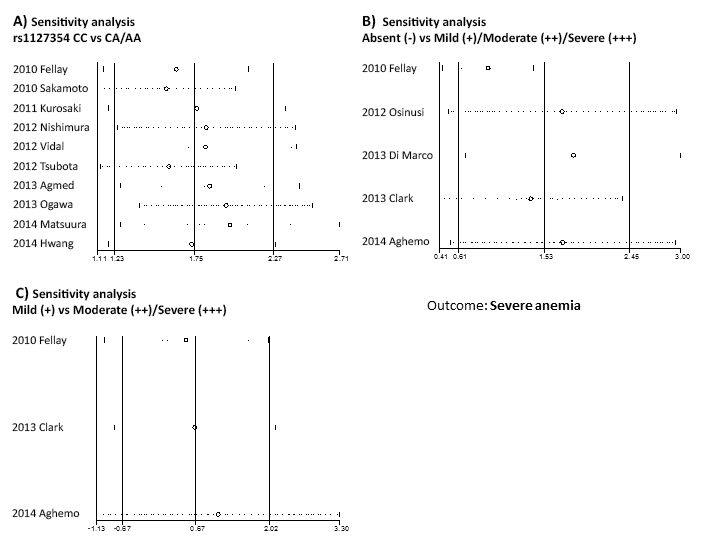


**Supplemental Figure 6.** Sensitivity analysis for studies included in the meta-analysis for ribavirin dose reduction or discontinuation: rs1127354 (A), haplotype absent (-) vs. mild (+)/moderate (++)/severe (+++) (B), haplotype mild (+) vs. moderate (++)/severe (+++) (C) and moderate (++) vs. severe (+++) (D). Sensitivity analyses were carried out to investigate the influence of any one study on the overall meta-analysis by sequential omission of individual studies.


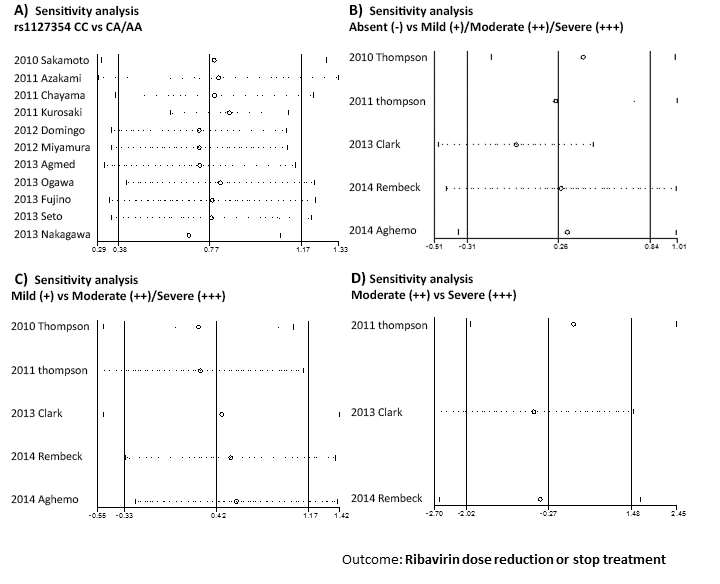

Supplement: Supplementary file 1 — 10.1186/s12967-015-0682-y Methodological quality of the studies included in the meta-analysis, which was assessed using a modified score based in the Newcastle–Ottawa Scale (Stang A. Critical evaluation of the Newcastle–Ottawa scale for the assessment of the quality of nonrandomized studies in meta-analyses. Eur J Epidemiol 2010;25:603–605). (see Supplemental Box 2). Table S2. Meta-regression analysis for hemoglobin decline (A–C), severe anemia (D) and ribavirin dose reduction or discontinuation (E) according to ITPA polymorphisms. Box 1. Description of rs1127354/rs7270101 haplotype: ITPase deficiency ranged from absent (-) (representing wild-type activity) to mild (+), moderate (++) or severe (+++) (8, 10). Box 2. Methodological criteria used to evaluate the quality of the studies included in the meta-analysis. This score was adapted from Newcastle–Ottawa Scale (Stang A. Critical evaluation of the Newcastle-Ottawa scale for the assessment of the quality of nonrandomized studies in meta-analyses. Eur J Epidemiol 2010;25:603–605). Figure S1. Publication bias for the rs1127354 studies included in the meta-analysis for hemoglobin decline(A), severe anemia (B) and ribavirin dose reduction or discontinuation (C) according both to Begg’s funnel plots and Egger’s test. Abbreviations: Coef., asymmetry regression coefficient; Std.Err., standard error; t, statistic; P>|t|, significance; and CI, confidence interval. Coefficients correspond to the intercept value in the regression equation, which estimates the asymmetry of the funnel plot. Positive values (Coef.> 0) indicate higher levels of effect size in studies with smaller sample sizes. Figure S2. Publication heterogeneity for studies included in the meta-analysis for hemoglobin decline: rs1127354 (A), rs7270101 (B), rs6051702 (C); severe anemia: haplotype rs1127354CC / rs7270101AA (D); and ribavirin dose reduction or discontinuation: rs1127354 (E); according to Galbraith’s plots. Abbreviations 1/s.e, precision; b/s.e, stan [file 12967_2015_682_MOESM1_ESM.doc]
